# Multiple Reassortment Events in the Evolutionary History of H1N1 Influenza A Virus Since 1918

**DOI:** 10.1371/journal.ppat.1000012

**Published:** 2008-02-29

**Authors:** Martha I. Nelson, Cécile Viboud, Lone Simonsen, Ryan T. Bennett, Sara B. Griesemer, Kirsten St. George, Jill Taylor, David J. Spiro, Naomi A. Sengamalay, Elodie Ghedin, Jeffery K. Taubenberger, Edward C. Holmes

**Affiliations:** 1 Department of Biology, Center for Infectious Disease Dynamics, The Pennsylvania State University, University Park, Pennsylvania, United States of America; 2 Fogarty International Center, National Institutes of Health, Bethesda, Maryland, United States of America; 3 Department of Global Health, School of Public Health and Health Services, The George Washington University, Washington, D.C., United States of America; 4 Wadsworth Center, New York State Department of Health, Albany, New York, United States of America; 5 The J. Craig Venter Institute, Rockville, Maryland, United States of America; 6 Institute for Genome Sciences, University of Maryland School of Medicine, Baltimore, Maryland, United States of America; 7 Division of Infectious Diseases, University of Pittsburgh, Pittsburgh, Pennsylvania, United States of America; 8 Laboratory of Infectious Diseases, National Institute of Allergy and Infectious Diseases, National Institutes of Health, Bethesda, Maryland, United States of America; University of Wisconsin-Madison, United States of America

## Abstract

The H1N1 subtype of influenza A virus has caused substantial morbidity and mortality in humans, first documented in the global pandemic of 1918 and continuing to the present day. Despite this disease burden, the evolutionary history of the A/H1N1 virus is not well understood, particularly whether there is a virological basis for several notable epidemics of unusual severity in the 1940s and 1950s. Using a data set of 71 representative complete genome sequences sampled between 1918 and 2006, we show that segmental reassortment has played an important role in the genomic evolution of A/H1N1 since 1918. Specifically, we demonstrate that an A/H1N1 isolate from the 1947 epidemic acquired novel PB2 and HA genes through intra-subtype reassortment, which may explain the abrupt antigenic evolution of this virus. Similarly, the 1951 influenza epidemic may also have been associated with reassortant A/H1N1 viruses. Intra-subtype reassortment therefore appears to be a more important process in the evolution and epidemiology of H1N1 influenza A virus than previously realized.

## Author Summary

The periodic occurrence of influenza epidemics in humans caused by viruses of the A/H1N1 subtype remains a key question in viral epidemiology and evolution and a major issue for public health. Since the first documentation of A/H1N1 in humans in 1918, this virus has been associated with a variety of epidemics and influenza vaccine failures. Using 71 representative whole-genome sequences of A/H1N1 influenza virus sampled between 1918 and 2005, we show that reassortment occurs frequently throughout the evolutionary history of this virus. Critically, two of these reassortment events appear to be associated with particularly severe epidemics, those of 1947 and 1951. Our analysis reveals that the virus associated with the 1947 epidemic was composed of genome segments with differing phylogenetic histories, suggesting that this virus was created through an intra-subtype reassortment event. Notably, of the two main antigenic proteins, the segment encoding the HA (hemagglutinin) is related to isolates circulating in a later time period, while the NA (neuraminidase) is related to earlier sampled isolates. This explains previous observations that the HA circulating at this time exhibited extensive antigenic drift while the NA appeared to be conserved. In addition, a virus likely associated with the 1951 epidemic also appears to have been generated by a reassortment event. Overall, our findings suggest that reassortment is an important factor in the long-term evolution of influenza A virus, including the periodic emergence of epidemic viruses. However, to more fully capture the evolutionary history of this important virus, additional sequencing of influenza viruses from earlier time periods is clearly needed.

## Introduction

Influenza A viruses of the H1N1 subtype, which circulated in humans from 1918–1957, and then again from 1977 to the present day, have had a significant epidemiological impact in humans. The most debated evolutionary question relating to this virus is how, and from where, A/H1N1 emerged in such a virulent form in 1918 to kill 20–50 million humans in the global influenza pandemic at the time of World War I, arguably the most severe single disease event in history [1],[2]. Perhaps as perplexing, however, is the evolutionary pattern of A/H1N1 influenza viruses following the 1918 pandemic, which is marked by a series of highly unusual occurrences, including the ‘pseudo-pandemic’ of 1947 [3] and several other severe epidemics in the 1920's through the 1950's, a twenty-year disappearance and sudden reappearance in 1977, and cycles of alternating dominance with the H3N2 influenza A virus subtype ever since.

Following the 1918 pandemic, the A/H1N1 influenza virus continued to circulate in humans, causing seasonal epidemics of varying severity [4] and also in swine, as ‘classical’ swine influenza [5]. In the post-pandemic period, epidemiologically severe outbreaks occurred in 1928–1929, 1932–1933, 1936–1937, and 1943–1944 in the United Kingdom [4] and the United States [6]. In 1947, the A/H1N1 virus underwent a major antigenic change that caused a total vaccine failure [7]. The virus was globally distributed much like a pandemic virus, but mortality was relatively low [8]. The virus was renamed ‘A-prime’ based on its antigenic divergence [9] from the previously characterized human A/H1N1 viruses of the early 1940's, although subsequent sequence analysis showed that these 1947 viruses were still of the A/H1N1 subtype, yet with numerous nucleotide and amino acid differences in antigenic regions of the hemagglutinin (HA) [10]. However, the evolutionary and epidemiological processes that precipitated such extensive divergence are currently unclear.

Another unusually severe A/H1N1 epidemic occurred in 1950–1951 [11], in which mortality levels in the United Kingdom and Canada exceeded those of both the 1957 and 1968 pandemics, again without a change in antigenic subtype [12]. In 1957, the A/H1N1 virus disappeared and was replaced by a novel H2N2 reassortant virus [13]. The A/H1N1 virus then resurfaced in 1977 after a twenty-year disappearance, causing an epidemic in children who lacked antibodies from prior exposure [14]–[17]. However, this emergent A/H1N1 did not replace the dominant H3N2 subtype [18], so that A/H1N1 and H3N2 have co-circulated to the present day. Although H3N2 has caused the majority of influenza A virus infections in recent decades, H1N1 periodically predominates during milder epidemic seasons [19]. Three of the past ten influenza seasons in the United States have been dominated by A/H1N1, all of which were mild and did not exceed the epidemic threshold for the proportion of deaths attributed to pneumonia and influenza (for example ref. [20]). How cross-immunity and other mechanisms dictate the cyclical interplay between the A/H1N1 and A/H3N2 subtypes in humans remains a major epidemiological question.

The role played by segmental reassortment in the evolution of A/H1N1 is also unclear. It is well established that reassortment between influenza isolates from different host species can generate viruses with pandemic potential. As case in point, reassortment between avian and human influenza A viruses generated the novel H2N2 and H3N2 strains that caused global human pandemics in 1957 and 1968, respectively [13],[21]. In addition, inter-subtype reassortment has been detected between co-circulating A/H1N1 and A/H3N2 viruses [22],[23], occasionally generating hybrid A/H1N2 viruses [24]. More recently, reassortment among influenza A viruses within the H3N2 subtype has been shown to generate both antigenically and genetically novel viruses, including those associated with vaccine strain mismatches [25]. To date, however, few studies of reassortment frequency in human A/H1N1 influenza viruses have been undertaken.

The A/H1N1 subtype is thought to experience less rapid antigenic evolution (‘antigenic drift’) than viruses of the A/H3N2 subtype, as reflected by the relatively infrequent need to update the A/H1N1 component of the human influenza vaccine [26],[27]. Whereas the A/H3N2 component of the influenza vaccine has been changed four times over the past seven years to account for frequent antigenic drift in this subtype, the A/New Caledonia/22/1999 (H1N1) strain has been used in the vaccine in each season from 2000–2001 to 2006–2007. This lower rate of antigenic drift in A/H1N1 presumably relates to reduced selection pressures, as reflected by the lower rates of nonsynonymous (d_N_) to synonymous substitutions (d_S_) per site (depicted in the ratio d_N_/d_S_) in A/H1N1 compared to A/H3N2 [28]. Understanding why A/H1N1 and A/H3N2 differ in their evolutionary and epidemiological dynamics remains a critical research question.

Herein, we undertook an expansive analysis of long-term evolutionary patterns in A/H1N1 influenza A viruses, using 71 whole-genome sequences (major coding regions) sampled between 1918 and 2006 and representing 17 different countries on five continents. As our focus is on revealing the extent of intra-subtype reassortment within A/H1N1, and particularly how reassortment events might relate to large-scale epidemiological patterns, we inferred phylogenetic trees for each individual gene segment and determined the extent and pattern of topological incongruence among them [25].

## Results

### Phylogenetic analysis of A/H1N1 genome sequences from 1918–2006

**Figure 1:**
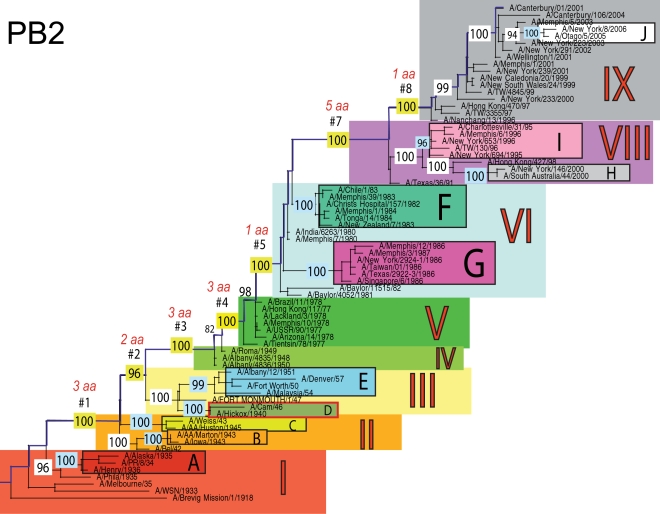
Phylogenetic relationships of the PB2 gene of A/H1N1 influenza viruses (n = 71) sampled globally from 1918–2006, estimated using a maximal likelihood (ML) method. All branch lengths are drawn to a scale of nucleotide substitutions per site. The tree is rooted using the oldest sequence, A/Brevig Mission/1/1918, and temporally ordered with the oldest isolates falling at the bottom left of the phylogeny and the newest isolates at the top right. Main phylogenetic sections that are separated by long trunk branches are numbered I–IX and colored as follows: section I is red, section II is orange, section III is yellow, section IV is light green, section V is dark green, section VI is light blue, section VII is absent due to the position of clade G within section VI (would otherwise be blue), section VIII is purple, and section IX is gray. The trunk branches separating these sections are labeled #1–#8 and highlighted in dark blue, with the bootstrap value supporting this branch highlighted in yellow and the number of amino acid changes that occur along each branch appearing above in red font. Ten main clades are labeled A–J, with supporting bootstrap values in light blue, and are colored as follows: clade A is dark red, clade B is orange, clade C is yellow, clade D is olive green, clade E is light blue, clade F is turquoise, clade G is fuchsia, clade H is gray, clade I is pink, and clade J is white. Clades that have undergone reassortment are outlined in red (in this case Clade D).

The phylogenetic trees inferred for all eight genome segments of 71 A/H1N1 viruses reveal a strong temporal structure, comprising a main trunk lineage that links viruses from successive epidemics, and short, transient, side branches [26],[29] (Figures 1–8). The topologies of all eight phylogenies also show generally similar evolutionary patterns. In particular, all trees can be divided into nine distinct topological sections, which we define as clusters of viruses that are separated by unusually long trunk branches (indicating extensive genetic divergence) with strong (usually 100%) bootstrap support (sections I–IX; trunk branches #1–#8). Although reassortment occasionally causes the movement of some viruses from one section into another (see below), for the most part each section on each tree contains the same set of viruses (Table S1). Section numbers increase chronologically on the trees: on the NP phylogeny, for example, section I contains isolates from 1918–1935, section II from 1942–1945, section III from 1940–1947, section IV from 1948–1957, section V from 1977 to 1978, section VI from 1980–1983, section VII from 1986–1987, section VIII from 1991–2000, and section IX from 1999–2006. As well as dividing the evolution of A/H1N1 into temporal sections, we were also able to identify ten distinct clades of viral isolates, each of which shares a unique common ancestor, supported by high (>70%) bootstrap values. These clades are denoted A to J in Figures 1–8.

**Figure 2 ppat-1000012-g002:**
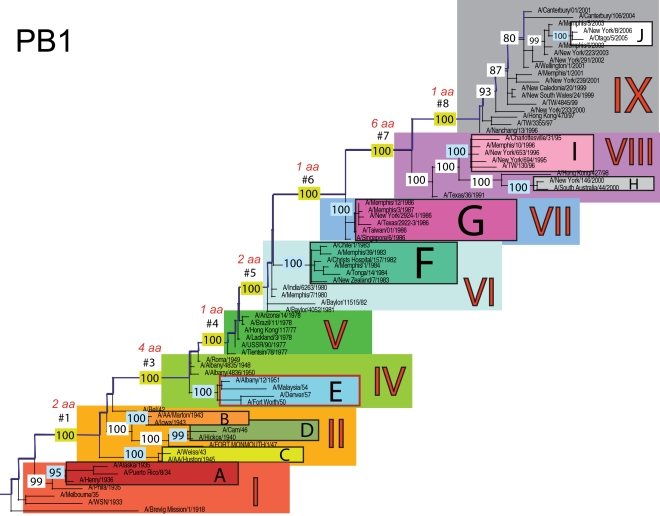
Phylogenetic relationships of the PB1 gene of A/H1N1 influenza viruses (n = 71) sampled globally from 1918–2006, estimated using ML. Rooting, scale, and color scheme are the same as those used in Figure 1.

**Figure 3 ppat-1000012-g003:**
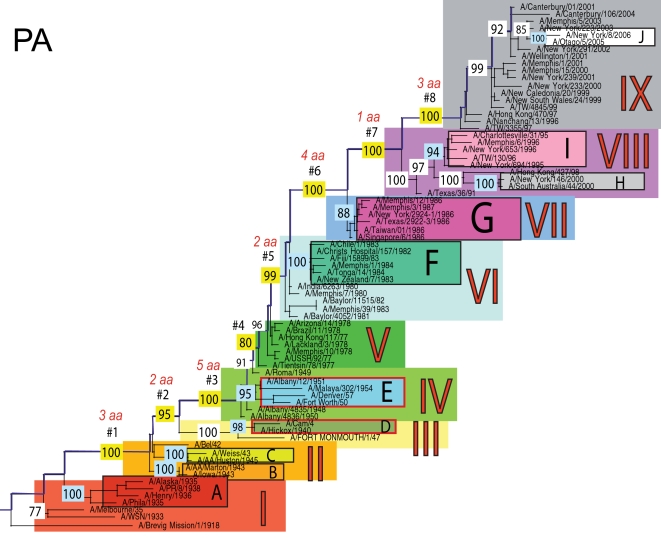
Phylogenetic relationships of the PA gene of A/H1N1 influenza viruses (n = 71) sampled globally from 1918–2006, estimated using ML. Rooting, scale, and color scheme are the same as those used in Figure 1.

**Figure 4 ppat-1000012-g004:**
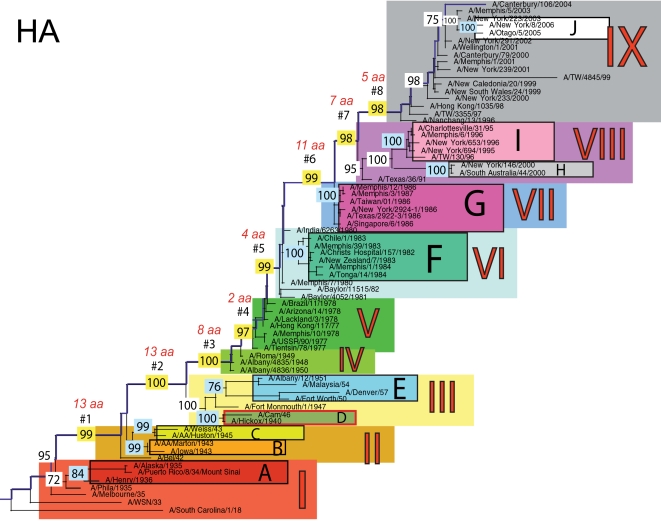
Phylogenetic relationships of the HA gene of A/H1N1 influenza viruses (n = 71) sampled globally from 1918–2006, estimated using ML. Rooting, scale, and color scheme are the same as those used in Figure 1.

**Figure 5 ppat-1000012-g005:**
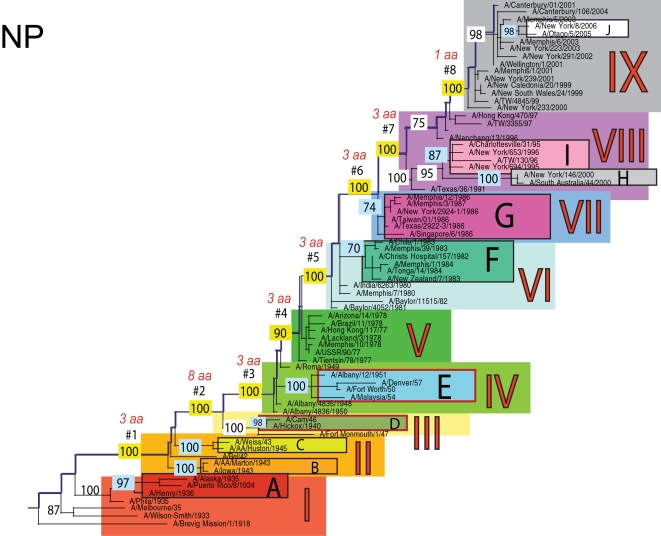
Phylogenetic relationships of the NP gene of A/H1N1 influenza viruses (n = 71) sampled globally from 1918–2006, estimated using ML. Rooting, scale, and color scheme are the same as those used in Figure 1.

**Figure 6 ppat-1000012-g006:**
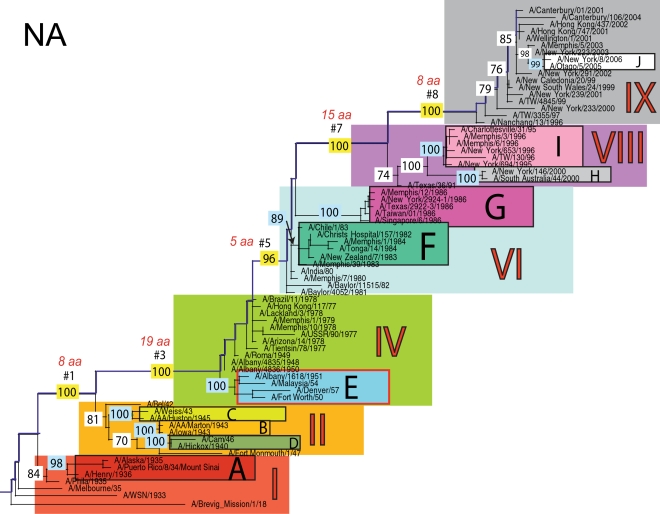
Phylogenetic relationships of the NA gene of A/H1N1 influenza viruses (n = 71) sampled globally from 1918–2006, estimated using ML. Rooting, scale, and color scheme are the same as those used in Figure 1.

**Figure 7 ppat-1000012-g007:**
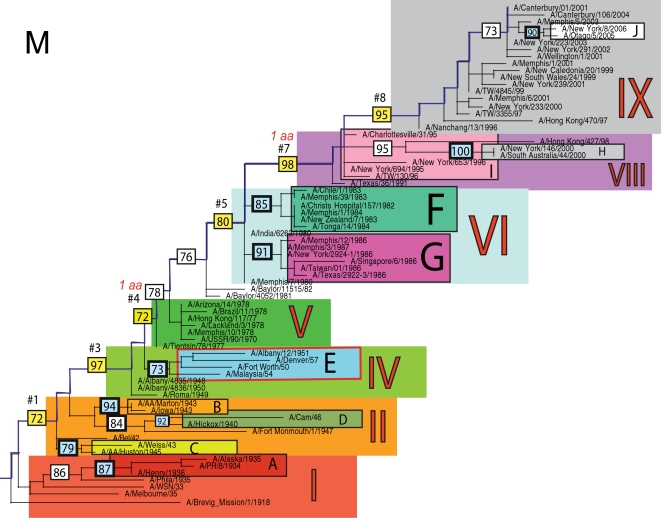
Phylogenetic relationships of the M1 gene of A/H1N1 influenza viruses (n = 71) sampled globally from 1918–2006, estimated using ML. Rooting, scale, and color scheme are the same as those used in Figure 1.

**Figure 8 ppat-1000012-g008:**
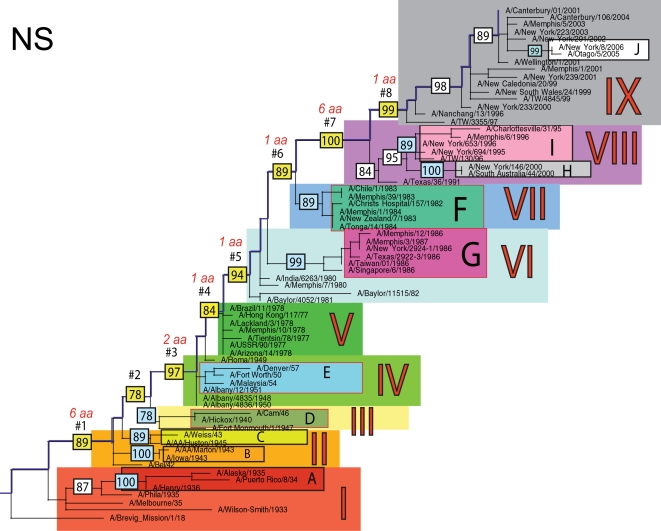
Phylogenetic relationships of the NS1 gene of A/H1N1 influenza viruses (n = 71) sampled globally from 1918–2006, estimated using ML. Rooting, scale, and color scheme are the same as those used in Figure 1.

Occasionally, two sections on a given tree merge into a single section due to the absence of the trunk branch that separates these sections on other segment phylogenies and, in some instances, the by action of reassortment. For example, in the PB1, NA, and M segments, sections II and III are merged into a single section (II) due to the close phylogenetic relationship of clades B, C, and D as a result of reassortment. Similarly, sections VI and VII are merged into a single VI section for the PB2, NA, and M gene segments, as clades F and G are positioned closely together on these three phylogenies (in the absence of trunk branch #6). Sections IV and V are also merged on the NA phylogeny.

The most frequent merging of sections occurs on the NA phylogeny; here, sections III, V, and VII are absent such that the NA phylogeny is comprised of only six of nine sections, and which results in the very long trunk branches #3 and #7. These two branches are especially notable on the NA tree in that they are characterized by an unusually high number of amino acid changes, reflecting the large evolutionary distance between sections II and IV (19 amino acid changes) and sections VI and VIII (15 amino acid changes) (Table 1). In marked contrast, no sections of the HA phylogeny are merged, resulting in a tree in which evolutionary change is more evenly distributed across all eight trunk branches. Across the viral genome as a whole, the greatest number of amino acid changes occurs along the main trunk lineages of the HA tree (n = 63), followed by the NA tree (n = 55), strongly supporting the long-term action of immune selection (antigenic drift) on these glycoproteins.

**Table 1:** Number of amino acid changes occurring along the main trunk branches of phylogenetic trees of each genome segment of A/H1N1 virus.

Branch	PB2	PB1	PA	HA	NP	NA	M1	NS1	Total
Branch #1	3	2	3	13	3	8	0	6	38
Branch #2	2	-	2	13	8	-	0	0	25
Branch #3	3	4	5	8	3	19	0	2	44
Branch #4	3	1	0	2	3	-	1	1	11
Branch #5	1	2	2	4	3	5	0	1	18
Branch #6	-	1	4	11	3	-	-	1	20
Branch #7	5	6	1	7	3	15	1	6	44
Branch #8	1	1	3	5	1	8	0	1	20
*Total*	*18*	*17*	*20*	*63*	*27*	*55*	*2*	*18*	*220*

Branch numbers correspond to those described in Figures 1–8. Dashes (-) refer to branches that are absent on a given phylogeny.

The smallest number of amino acid changes occurs along branch #4, which connects isolates from the 1950's (section IV) with those from the 1970's (section V). Thus, little A/H1N1 evolution is evident over the twenty-year period of the virus's global disappearance [30], supporting earlier suggestions that this subtype was most likely accidentally reintroduced into human circulation from a laboratory environment [3],[31]. Notably, our analysis indicates that the influenza viruses that re-emerged in the 1970's were more closely related in all gene segments to a group of viruses sampled from the late 1940's, in particular to isolate A/Roma/1949, supporting earlier serological and partial sequence analyses [16],[30],[32]

### Multiple reassortment events within A/H1N1

In general, most of the ten clades A–J fall within the same topological section in each of the segment phylogenies. For example, on all eight phylogenies, clade A is positioned within section I, clades B and C fall in section II, clades H and I are contained in section VIII, and clade J is found within section IX. In contrast, clades D, E, F, and G have markedly different topological (section) positions among segments, revealing the past history of reassortment (Figures 1–8).

**Figure 9:**
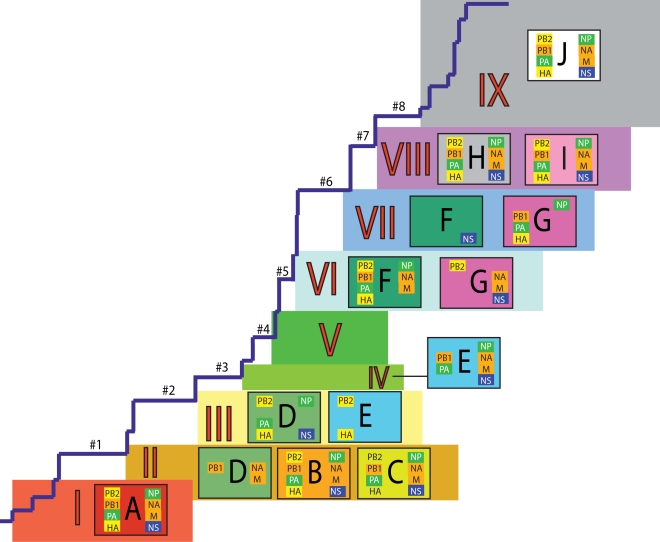
Schematic representation of the phylogenetic patterns of all eight A/H1N1 influenza virus genomes used in this study (Figures 1–8). The main trunk (back-bone) lineage is taken from the HA phylogeny, with the nine sections I–IX and long trunk braches #1–8 corresponding to their respective positions on HA phylogeny and similarly color-coded. Clades A–J are also color-coded as in Figures 1–8. Each clade is positioned within a given phylogenetic section for all viral genome segments contained within the box. Viral genome segments are color-coded to show similarities in the phylogenetic patterns between multiple segments: PB2 and HA follow similar phylogenetic patterns and are colored in yellow boxes; PB1, NA, and M are phylogenetically similar and are shaded in orange boxes; PA and NP are in green boxes; NS follows a pattern unique to itself and is alone colored in a dark purple box.

A summary of the differing phylogenetic patterns of each viral genome segment, highlighting the occurrence of reassortment, is provided in Figure 9. Most clades (A, B, C, H, I, J) occupy a single position on this phylogenetic representation and are clearly non-reassortant. In contrast, clades D, E, F, and G occupy different topological sections for different genome segments, reflecting a pattern of phylogenetic incongruence caused by whole-genome reassortment. Clade D falls into section II for segments PB1, NA, and M, but into section III for segments PB2, PA, HA, NP, and NS. Clade E falls into section III for segments PB2 and HA, but into section IV for PB1, PA, NP, NA, M, and NS. Clade F falls into section VI for all segments except NS, in which clade F falls into section VII through a reversal of positions with clade G, which is found in section VI. Such phylogenetic incongruences provide strong evidence for intra-subtype reassortment.

Due to the action of reassortment, Clade D, containing viruses sampled between 1940 and 1947, occupies a variety of phylogenetic positions. This topological movement reflects how genomic segments are related to a variety of clades sampled from different time periods (Figures 1–9). For three genome segments (PB1, NA, and M), clade D is positioned with clades B and C in section II, revealing its close phylogenetic relationship to viral isolates sampled from the early 1940's in these segments. Thus, our analysis strongly suggests that at least three distinct clades of A/H1N1 viruses must have co-circulated during the 1940's (B,C,D); phylogenetic support for these clades is evident in all eight gene segments. Alternatively, for the majority of gene segments (PB2, PA, HA, NP, and NS), clade D falls within section III, either individually (PA, NP, and NS) or with clade E (PB2 and HA). Thus, for these five gene segments, clade D is no longer closely related to viral isolates sampled between 1943–1945 in clades B and C, but rather to later-sampled isolates. Such marked topological incongruence across the viral genome provides strong evidence for the action of reassortment in which viruses from clade D acquired genetic material from other co-circulating clades that have yet to be sampled.

Clade E (representing viruses isolated in 1950–1957) similarly exhibits different topological patterns across the eight segment phylogenies (Figures 1–9). For six of the gene segments (PB1, PA, NP, NA, M, and NS), clade E is positioned in section IV along with three viral isolates from 1948–1950: A/Roma/1949, A/Albany/4835/1948, and A/Albany/4836/1950. However, for PB2 and HA, clade E is positioned in section III along with clade D isolates from an earlier era, 1940–1947. That the PB1, PA, NP, NA, M and NS segments from clade E are more closely related to viral isolates from the latter part of the 1940's and early 1950's strongly suggests that clade E viruses were also generated by reassortment.

The action of reassortment is also apparent from the variable phylogenetic positions of clades F and G. For seven of eight gene segments (PB2, PB1, PA, HA, NP, NA, and M), clade F is positioned within section VI. In contrast, clade F falls into section VII for the NS segment due to a topological reversal between clades F and G, indicative of reassortment. Clade G is also found in various topological positions, suggesting further reassortment, although phylogenetic resolution in this portion of the tree is insufficient to infer the action of reassortment with any statistical certainty. For half of the viral genome (PB1, PA, HA, and NP), clade G clearly falls into section VII, topologically distinct from clade F in section VI. In contrast, for the remainder of the genome, clade G is either positioned with clade F in section VI (PB2, NA, M), or occupies section VI in isolation (NS).

Overall, we can characterize four distinct evolutionary patterns within the genome of A/H1N1 viruses (Figure 9). The PB2 and HA phylogenies (represented by yellow boxes in Figure 9) are both characterized by a pattern in which there has been a single reassortment event involving clade D. PB1, NA, and M (orange boxes) are characterized by a second pattern involving the reassortment of clade E. Third, the PA and NP phylogenies (green boxes) display two reassortment events involving both clades D and E. Lastly, the evolutionary history of the NS segment (dark purple boxes) includes at least three reassortment events involving clades D, E, F, and G.

## Discussion

Although the eight genome segments of H1N1 influenza A viruses exhibited generally congruent evolutionary patterns during their circulation in the twentieth century, we found strong phylogenetic evidence for several distinct reassortment events affecting specific segments. Analyses of the complete genomes of a large number of recently sampled human H3N2 influenza A viruses have demonstrated the co-circulation of multiple distinct clades and frequent intra-subtype reassortment events among them [25],[33]. Although far fewer A/H1N1 viral genomes from the 1930's–1950's are available for analysis, even with this limited sample distinct co-circulating clades and several intra-subtype reassortment events are apparent, suggesting that the evolutionary complexity of recent A/H3N2 viruses was likely recapitulated in A/H1N1 viruses from this era. Further, while adaptation to growth in embryonated chicken eggs is likely to influence patterns of viral evolution in early sampled isolates, and particularly in the HA [34], such bias will largely affect tip (terminal) rather than trunk branches of phylogenetic trees [34], and so will have no major bearing on the results presented here.

Interestingly, in two cases these observed reassortment events occurred concurrently with the unusual influenza epidemics of 1947 and 1951. In both these years influenza viruses emerged that displayed certain characteristics of pandemic viruses, including unusually high morbidity and mortality impact, but which did not acquire new gene segments through reassortment with other influenza virus subtypes. Hence, our analysis is compatible with intra-subtype reassortment events involving multiple segments playing a role in the genesis of these unusual epidemic viruses, a phenomenon that was only recently demonstrated to occur among influenza viruses of the A/H3N2 subtype [25].

The most notable observation from our study is that the clade D reassortment event appears to coincide with the unusually severe post-World War II influenza epidemic of 1947, which caused a total influenza vaccine failure worldwide although with relatively low mortality [7]. Previous analyses revealed that the HA1 region of the hemagglutinin of these 1947 epidemic influenza isolates, including A/Fort Monmouth/1/47, were highly divergent from those of the less virulent isolates sampled between 1943–1945, including A/Weiss/43 and A/Marton/43 [10], and which are represented by the co-circulating clades B and C in this study (Figures 1–8). Based on this marked antigenic change, Kilbourne *et al.* [10] suggested that the 1947 epidemic viruses did not evolve directly from the 1943–1945 viruses that were dominant earlier, but rather may have been derived from a minor A/H1N1 clade that was circulating undetected. In our analysis, the A/Fort Monmouth/1/47 epidemic virus is closely related to clade D viruses in each of the eight segments (and is even included within clade D on the PA, NP, M, and NS phylogenies), and therefore follows the exact same evolutionary pattern as the reassortant clade D. Thus, this phylogenetic analysis suggests that the 1947 epidemic virus was generated by a major reassortment event, in which the PB1, NA, and M segments from A/H1N1 viruses that were predominant from 1943–1945 were combined with novel PB2, PA, HA, NP, and NS gene segments that were perhaps derived from a minor A/H1N1 clade that unfortunately was not detected by surveillance efforts at the time. It is unclear, however, whether the impact of the 1947 epidemic was due entirely to the evolutionary novelty provided by an antigenically distinct HA segment acquired through reassortment, or whether mutational changes in PB2, PA, NP, and/or NS in the new viral genomic context also played important roles in altering virus virulence and transmissibility. It is also uncertain whether retention of an older NA antigen may have mitigated its effect on mortality, as has been suggested in the case of the 1968 pandemic, in which the severity of infections was moderated by the human population's residual antibody to the retained N2 surface protein [35]. Indeed, determining the epistatic interactions among the segments of the influenza virus genome and their roles in viral transmissibility and virulence remains a major research goal.

Our analysis also suggests, more tentatively, that the virus responsible for the unusually severe 1951 epidemic in some geographic regions may have been generated by a genomic reassortment event. Based on the phylogenetic movement of clade E, a major reassortment event clearly occurred in the early 1950's to create a virus with novel PB1, PA, NP, NA, M, and NS gene segments in combination with older PB2 and HA genes that were closely related to those circulating in the 1940's. The extensive evolutionary change in six of the eight viral gene segments generated in this reassortment event may resolve the quandary over how a virus that displayed little antigenic drift in HA caused such a severe epidemic [11]. It has been previously suggested that the severity of the 1951 epidemic in the UK and Canada was related to the high transmissibility of the virus circulating in these countries, which perhaps resulted from enhanced viral replication within hosts [36]. Our finding that clade E retained its HA gene but acquired two polymerase genes – PB1 and PA – through reassortment suggests that these viruses indeed may have been antigenically similar but replicated with enhanced efficiency. However, it is not known whether the A/Fort Worth/1950 and/or A/Albany/12/1951 isolates contained within clade E were derived from the severe 1951 epidemic. Although both isolates were circulating at the time of the epidemic, and the A/Albany/12/1951 isolate (sampled during April 1951) originates in an area of the northeastern United States that experienced particularly high mortality [6], no influenza virus sequence that is known with certainty to have been sampled from the severe 1951 epidemic is available in the public domain. Additional sequencing of isolates from the 1950's, particularly from areas most affected by the epidemic, including the United Kingdom and Canada, is clearly required to evaluate the role of reassortment in generating the influenza viruses that caused this unusual epidemic.

While the epidemiological significance of the reassortment events documented here remains unresolved, particularly in the case of the 1951 epidemic, our analysis shows for the first time that large-scale intra-subtype reassortment events, involving all eight segments of the viral genome, have played an important role in the evolutionary history of the A/H1N1 virus.

## Materials and Methods

### Influenza viruses used in this study

All complete genome sequences of influenza A/H1N1 virus data were collected as part of the Influenza Genome Sequencing Project (http://www.niaid.nih.gov/dmid/genomes/mscs/influenza.htm) for the period 1918–2006 [37]. All sequence data were downloaded from the National Center for Biotechnology Information Influenza Virus Resource (http://www.ncbi.nlm.nih.gov/genomes/FLU/FLU.html). A total of 420, 419, 418, 480, 448, 547, 475, and 444 full-length A/H1N1 sequences were available for the PB2, PB1, PA, HA, NP, NA, M, and NS segments, respectively, the vast majority of which were collected from Australia, New Zealand or the United States during the period 1995–2005. After removing isolates for which not all nucleotide sequences for all eight genome segments were available, phylogenetically closely related sequences from the same year and location, and sequences acquired directly from swine, a total of 71 representative full-length influenza A/H1N1 virus sequences from 17 countries spanning five continents were used in the analysis. Full-length sequences were available for all segments except the HA gene of the A/Brevig Mission/1/1918 virus, for which a phylogenetically related virus sampled from the same year – A/South Carolina/1/18 – was used in its place for this segment. GenBank accession numbers for all sequences used in this study are listed in Table S1.

### Phylogenetic analysis

Sequence alignments were manually constructed for the major coding regions of each of the eight genomic segments: PB2 (2,277 nt), PB1 (2,271 nt), PA (2,148 nt), HA (1,698 nt), NP (1,494 nt), NA (1,407 nt), M1 (756 nt), and NS1 (690 nt). Because the small M2 and NS2 proteins are involved in overlapping reading frames, they were not included in the analysis.

Maximum likelihood (ML) phylogenetic trees were inferred for each of the eight genome segments sequences using the PAUP* package [38]. In each case, the best-fit model of nucleotide substitution was identified by MODELTEST [39] as the general reversible GTR+I+Γ_4_ model, with the frequency of each substitution type, proportion of invariant sites (I), and the gamma distribution of among-site rate variation with four rate categories (Γ_4_) estimated from the empirical data (parameter values available from the authors on request). In all cases TBR (tree bisection-reconnection) branch-swapping was then utilized to determine the optimal tree. A bootstrap resampling process (1,000 replications) using the neighbor-joining (NJ) method was used to assess the robustness of individual nodes on the phylogeny, incorporating the ML substitution model. Finally, fixed amino acid changes along major branches of the phylogeny were identified using the parsimony algorithm available in the MacClade program [40].

Viral clades were identified as clusters of isolates sharing a common ancestor with >70% bootstrap support on all eight phylogenies. Due to the comparatively low resolution of the M and NS phylogenies, itself a function of the short length and conserved nature of the M and NS segments, isolates from clades E and I formed clusters supported by bootstrap values that did not reach 70%. However, these isolates clearly clustered together across all segment phylogenies and so are depicted as a single clade for the sake of clarity and consistency.

## Supporting Information

Table S1 GenBank accession numbers and background information for 71 complete genome sequences of influenza A virus subtype H1N1 used in the phylogenetic analysis. For simplicity, accession numbers refer to the PB2 gene. Clade letters and section numbers correspond to those given in Figure 1. All genome sequences were downloaded from the Influenza Virus Resource available through GenBank (http://www.ncbi.nlm.nih.gov/genomes/FLU/FLU.html).
(0.13 MB DOC)
