# Supplementary material for: Multiple Reassortment Events in the Evolutionary History of H1N1 Influenza A Virus Since 1918
Source: PLoS Pathog. 2008 Feb 29;4(2):e1000012. doi: 10.1371/journal.ppat.1000012 (PMC2262849; doi:10.1371/journal.ppat.1000012)
Supplement: Table S1 — GenBank accession numbers and background information for 71 complete genome sequences of influenza A virus subtype H1N1 used in the phylogenetic analysis. For simplicity, accession numbers refer to the PB2 gene. Clade letters and section numbers correspond to those given in Figure 1. All genome sequences were downloaded from the Influenza Virus Resource available through GenBank (http://www.ncbi.nlm.nih.gov/genomes/FLU/FLU.html). (0.13 MB DOC) [file ppat.1000012.s001.doc]

**Table S1.** GenBank accession numbers and background information for 71 complete genome sequences of influenza A virus subtype H1N1 used in the phylogenetic analysis. For simplicity, accession numbers refer to the PB2 gene. Clade letters and section numbers correspond to those given in Figure 1.

All genome sequences were downloaded from the Influenza Virus Resource available through GenBank (<http://www.ncbi.nlm.nih.gov/genomes/FLU/FLU.html>).

|  | GenBank  Accession | Country of Isolation | Year of Isolation | Isolate | Clade | Section |
| --- | --- | --- | --- | --- | --- | --- |
| 1 | DQ208309 | USA | 1918 | A/Brevig Mission/1/1918 | n/a | I |
| 2 | J02179 | United Kingdom | 1933 | A/WSN/1933 | n/a | I |
| 3 | V00603 | Puerto Rico | 1934 | A/Puerto Rico/8/34 | A | I |
| 4 | CY009331 | Australia | 1935 | A/Melbourne/1935 | n/a | I |
| 5 | CY019962 | USA | 1935 | A/Alaska/1935 | A | I |
| 6 | CY020476 | USA | 1935 | A/Phila/1935 | n/a | I |
| 7 | CY020452 | ? | 1936 | A/Henry/1936 | A | I |
| 8 | CY013278 | USA | 1940 | A/Hickox/1940 | D | III |
| 9 | CY009283 | USA | 1942 | A/Bel/1942 | n/a | II |
| 10 | CY009459 | USA | 1943 | A/Weiss/1943 | C | II |
| 11 | CY020468 | USA | 1943 | A/Iowa/1943 | B | II |
| 12 | CY020292 | USA | 1943 | A/AA/Marton/1943 | B | II |
| 13 | CY021716 | USA | 1945 | A/AA/Huston/1945 | C | II |
| 14 | CY009603 | ? | 1946 | A/Cam/1946 | D | III |
| 15 | CY009619 | USA | 1947 | A/FortMonmouth/1/1947 | D | III |
| 16 | CY019954 | USA | 1948 | A/Albany/4835/1948 | n/a | IV |
| 17 | CY019978 | Italy | 1949 | A/Roma/1949 | n/a | IV |
| 18 | CY021708 | USA | 1950 | A/Albany/4836/1950 | n/a | IV |
| 19 | CY009339 | USA | 1950 | A/Fort Worth/1950 | E | III |
| 20 | CY021828 | USA | 1951 | A/Albany/12/1951 | E | III |
| 21 | CY009347 | Malaysia | 1954 | A/Malaysia/1954 | E | III |
| 22 | CY008995 | USA | 1957 | A/Denver/1957 | E | III |
| 23 | CY020580 | China | 1977 | A/Tientsin/78/1977 | n/a | V |
| 24 | DQ508894 | Russia | 1977 | A/USSR/90/1977 | n/a | V |
| 25 | CY009299 | Hong Kong | 1977 | A/Hong Kong/117/1977 | n/a | V |
| 26 | CY010875 | USA | 1978 | A/Memphis/10/1978 | n/a | V |
| 27 | CY020300 | Brazil | 1978 | A/Brazil/11/1978 | n/a | V |
| 28 | CY019970 | USA | 1978 | A/Arizona/14/1978 | n/a | V |
| 29 | CY020172 | USA | 1978 | A/Lackland/3/1978 | n/a | V |
| 30 | CY010915 | USA | 1980 | A/Memphis/7/1980 | n/a | VI/VII |
| 31 | CY020460 | India | 1980 | A/India/6263/1980 | n/a | VI/VII |
| 32 | CY021036 | USA | 1981 | A/Baylor/4052/1981 | n/a | VI/VII |
| 33 | CY021044 | United Kingdom | 1982 | A/Christ's Hospital/157/1982 | F | VI/VII |
| 34 | CY010371 | USA | 1982 | A/Baylor/11515/1982 | n/a | VI/VII |
| 35 | X15283 | Chile | 1983 | A/Chile/1/1983 | F | VI/VII |
| 36 | CY020196 | New Zealand | 1983 | A/New Zealand/7/1983 | F | VI/VII |
| 37 | CY020244 | USA | 1983 | A/Memphis/39/1983 | F | VI/VII |
| 38 | CY020492 | Tonga | 1984 | A/Tonga/14/1984 | F | VI/VII |
| 39 | CY021732 | USA | 1984 | A/Memphis/1/1984 | F | VI/VII |
| 40 | CY021740 | USA | 1986 | A/New York/2924-1/1986 | F | VI/VII |
| 41 | CY020572 | USA | 1986 | A/Texas/2922-3/1986 | G | VI/VII |
| 42 | DQ508870 | Taiwan | 1986 | A/Taiwan/01/1986 | G | VI/VII |
| 43 | CY020484 | Singapore | 1986 | A/Singapore/6/1986 | G | VI/VII |
| 44 | CY019108 | USA | 1986 | A/Memphis/12/1986 | G | VI/VII |
| 45 | CY019778 | USA | 1987 | A/Memphis/3/1987 | G | VI/VII |
| 46 | CY009323 | USA | 1991 | A/Texas/36/91 | n/a | VIII |
| 47 | AF398866 | USA | 1995 | A/Charlottesville/31/95 | I | VIII |
| 48 | CY015539 | USA | 1995 | A/New York/694/1995 | I | VIII |
| 49 | CY013820 | China | 1996 | A/Nanchang/13/1996 | n/a | IX |
| 50 | CY019794 | USA | 1996 | A/Memphis/6/1996 | I | VIII |
| 51 | DQ415283 | Taiwan | 1996 | A/TW/130/96 | I | VIII |
| 52 | CY010843 | USA | 1996 | A/New York/653/1996 | I | VIII |
| 53 | AF258524 | Hong Kong | 1997 | A/Hong Kong/470/97 | n/a | VIII |
| 54 | DQ415284 | Taiwan | 1997 | A/TW/3355/97 | n/a | IX |
| 55 | AF258525 | Hong Kong | 1998 | A/Hong Kong/427/98 | n/a | IX |
| 56 | CY017130 | Australia | 1999 | A/New South Wales/24/1999 | n/a | IX |
| 57 | DQ415285 | Taiwan | 1999 | A/TW/4845/99 | n/a | IX |
| 58 | DQ508854 | New Caledonia | 1999 | A/New Caledonia/20/1999 | n/a | IX |
| 59 | CY021756 | Australia | 2000 | A/South Australia/44/2000 | H | IX |
| 60 | CY000455 | USA | 2000 | A/New York/146/2000 | H | IX |
| 61 | CY002647 | USA | 2000 | A/New York/233/2000 | n/a | IX |
| 62 | CY010403 | New Zealand | 2001 | A/Canterbury/01/2001 | n/a | IX |
| 63 | CY020268 | USA | 2001 | A/Memphis/1/2001 | n/a | IX |
| 64 | CY003007 | USA | 2001 | A/New York/239/2001 | n/a | IX |
| 65 | CY011159 | New Zealand | 2001 | A/Wellington/1/2001 | n/a | IX |
| 66 | CY003311 | USA | 2002 | A/New York/291/2002 | n/a | IX |
| 67 | CY020252 | USA | 2003 | A/Memphis/5/2003 | J | IX |
| 68 | CY002695 | USA | 2003 | A/New York/223/2003 | J | IX |
| 69 | CY007474 | New Zealand | 2004 | A/Canterbury/106/2004 | n/a | IX |
| 70 | CY013580 | New Zealand | 2005 | A/Otago/5/2005 | J | IX |
| 71 | CY017378 | USA | 2006 | A/New York/8/2006 | J | IX |
